# Combatting the Caregiver Crisis: Health and Social Inequities for ADRD Caregivers with Disabilities

**DOI:** 10.1093/geroni/igaf122.3734

**Published:** 2025-12-31

**Authors:** Nicholas Mirin, Laurin Bixby, Joe Caldwell

**Affiliations:** Brandeis University, Waltham, Massachusetts, United States; Brandeis University, Waltham, Massachusetts, United States; Brandeis University, Waltham, Massachusetts, United States

## Abstract

As the population ages and the care workforce crisis persists, an increasing number of adults are becoming unpaid caregivers for aging parents or other family members with ADRD. While people with disabilities are often assumed to be on the receiving end of care, we estimate 36.4% of disabled adults are caregivers themselves, many of whom provide care to individuals with ADRD. Prior research documents disparities for people with disabilities and caregivers separately, but little attention has been given to the intersection of disability and caregiving. As such, disabled caregivers are largely left out of research and policy discussions related to the impact of ADRD on family caregivers. To address that literature gap, this study uses multivariate logistic regression and data from the 2021–2023 Behavioral Risk Factor Surveillance System (n = 310,722) to estimate health and social determinants of health risks as a function of the interaction between disability and ADRD caregiving status among unpaid caregivers, adjusting for demographic and household characteristics. Results indicate that disabled ADRD caregivers report poorer physical, mental health, and social support outcomes relative to non-disabled caregivers – including elevated risks of housing, food, and job insecurity, as well as significant barriers to transportation. We also find disabled ADRD caregivers disproportionately rely on public assistance programs such as SNAP. Taken together, these findings suggest disabled ADRD caregivers experience compounded risks of adverse health and SDH outcomes. Future policies must address the challenges experienced by family caregivers of adults with ADRD, particularly among those who have disabilities themselves.

